# A Novel Multiplex Diagnostic Algorithm for Developing an Optimal Patient-Tailored Therapy in Rheumatoid Arthritis Based on Molecular Stratification

**DOI:** 10.3390/ijms26125640

**Published:** 2025-06-12

**Authors:** Angeliki Margoni, Alice G. Vassiliou, Athanasios G. Papavassiliou

**Affiliations:** 1 Department of Biological Chemistry, Medical School, National and Kapodistrian University of Athens, 11527 Athens, Greece; angeliki.margoni@gmail.com; 2 First Department of Critical Care Medicine, Evangelismos Hospital, Medical School, National and Kapodistrian University of Athens, 10676 Athens, Greece; alvass75@gmail.com

Rheumatoid arthritis (RA) is a progressive and inflammatory autoimmune disease that displays high heterogeneity in etiology, clinical presentation, prognosis, and response to therapy. RA is a multifactorial polyarthritis that affects approximately 1% of the global population and is characterized by local inflammation and cartilage destruction. Several environmental factors are related to the triggering of the autoimmune response in the synovium, resulting in inflammation through the activation of T-cells, B-cells, and macrophages, as well as the release of cytokines and interleukins. Interleukin 1 (IL-1), IL-6, IL-17, and tumor necrosis factor-alpha (TNF-α) are the main inflammatory factors that promote joint damage [1,2]. Notwithstanding the wide range of therapeutic interventions, long-term complications still occur at different rates among different subtypes, provoking reduced mobility, chronic incapacity, and decreased life expectancy. Currently, the drug armamentarium of rheumatologists is amplified by biologic disease-modifying antirheumatic drugs (b-DMARDs), as well as glucocorticoids, non-steroidal anti-inflammatory drugs (NSAIDs), analgesics, and conventional synthetic (cs-DMARDS) therapies such as methotrexate (MTX). The administration of b-DMARDS as a second-line treatment has increased remission rates; however, it exhibits a similar rate of significant clinical improvement compared to the most-prescribed and cost-effective therapy—MTX [1,2]. The most commonly used b-DMARDs are TNF-α, IL-6, and IL-17 inhibitors, which are monoclonal antibodies with a broad range of effectiveness, ranging from remission to a total lack of response [1,2,3]. It is set in stone that diversity in molecular and genetic status prejudges drug sensitivity and disease progression, frequently resulting in therapeutic failure as no reliable predictive index exists. Therefore, it is vital to redefine the diagnostic enquiry of RA with a spherical approach based on molecular diagnosis and classification.

Rheumatologists aim to achieve a long-term stable remission or a low disease activity to prevent irreversible joint damage. Their primary goal is to adjust the therapeutic strategy in an economically sustainable way and to maintain remission with minimum complications. Despite the rapidly expanding horizon of precision medicine, RA diagnosis is still based on clinical criteria and only in 2010, were rheumatoid factor (RF) and anti-cyclic citrullinated peptide (anti-CCP) antibodies included in revised “RA classification criteria” despite their low specificity and sensitivity [4]. The “trial and error” approach, particularly with long-term b-DMARDS therapy, leads to an elevated risk of severe infections due to immunosuppression, variability in therapeutic effectiveness, and high economic cost. In this context, numerous clinical studies have focused on integrating clinical and biochemical markers into a widely recognized algorithm that facilitates the establishment of the optimal therapeutic dose, ameliorates effectiveness, and prevents clinical recurrence. Nevertheless, the Disease Activity Score of 28 joints (DAS28), serum levels of C-reactive protein (CRP), RF, and positivity for anti-CCP antibodies (ACPA) was proven to have a weak predictive ability and failed to restrain therapeutic failure [1,3]. However, APCA is an independent risk factor for RA development, classifying RA in positive and negative APCA subtypes, which differ in genetic profile and clinical progression [5]. The lack of an accurate prognostic marker leads to numerous imaging procedures, unnecessary laboratory tests, ineffective and expensive biological regimens, and frequent hospitalization. Given the severe impact on public health and the health economy, clinical research should be amplified towards the development of a reliable, less invasive, and cost-effective index that stratifies patients according to their molecular signature, suggesting an individualized therapeutic strategy. This evidence-based immunomodulation therapy approach diminishes the risk of long-term complications and drug side effects, eliminating drug resistance compared to empirical therapy. Furthermore, the measurement of multiple biomarkers that intervene with systematic inflammation and immune deregulation enhances research for the development of novel cell therapies. Towards the goal of precision medicine, a diagnostic model that incorporates molecular biomarkers should be evaluated in the clinical setting, concerning its prognostic value and clinical significance.

Recent advances in molecular technologies highlight the potential role of genomics and proteomics in precise diagnosis, classification, personalized therapy, as well as in relation to follow-up protocols. Given the high genetic contribution in RA that reaches ~60%, genomics may also be useful in terms of prevention by implicating patients with a strong genetic predisposition, who could benefit from prevention measures or tailored treatment [5,6]. Early initiated therapy to the high-risk population improves the response to drugs and augments the remission rate compared to delayed therapy. It has been well-established that the major histocompatibility complex (MHC) human leukocyte antigens (HLAs) locus on chromosome 6 plays a key role in encoding molecules that foster autoimmune responses. Further genetic testing has revealed over 100 different loci of RA susceptibility, whereas HLA haplotypes and specifically HLA-DRB1 alleles (DR4 and DR1), are predominant. However, RA is a heterogenic and polygenic disease, as both HLA and non-HLA genes are related to different types of RA, disclosing the rising diagnostic value of molecular methods, such as next-generation sequencing (NGS) [6]. NGS results revealed that certain polymorphisms and gene alterations are associated with a higher response to MTX, while others confer a higher risk for therapeutic failure. Thus, identifying the genetic signature may predict the response to cs-DMARDs or the need to include biologic therapy. Moreover, multiple gene loci and single-nucleotide polymorphisms (SNPs) are associated with an increased response to anti-TNF-α, allowing rheumatologists to reduce the dose and duration of treatment with safety. Circulating levels of micro(mi)RNA-19b-3p in whole blood present fluctuations during RA development, whilst reduced levels reflect a response to therapy even within 3 months of Janus kinase (JAK) inhibitor (JAKI) treatment [5,6,7]. Additionally, miRNA-19b-3p levels seem to have a statistically significant decline when RA is effectively treated, which corresponds to a clinical improvement and a reduction in DAS28 score. Accordingly, miRNA levels are a promising prognostic blood biomarker that may serve in RA surveillance and the early diagnosis of drug resistance [7,8,9].

Proteomics is a dynamic study of multiple proteins that are expressed under certain times and conditions, reflecting the progression of inflammation and tissue damage. The gold standard method for proteomic analysis is automated multiplexed immunoassays in serum samples, following a simple blood collection [6,10]. Research has focused on the discovery of novel biomarkers with clinical significance on early diagnosis, classification, accurate prognosis, and follow-up. Certain markers, such as the 14-3-3 protein, anti-peptidylarginine deaminase (anti-PAD), and anti-mutated citrullinated vimentin (anti-MCV) antibodies are associated with radiographic progression, while anti-carbamylated protein (anti-CarP) antibodies increase their diagnostic and prognostic value in combination with RF/ACPA [11]. The most promising multi-biomarker disease activity (MBDA) score depicts the serum level of 12 different proteins, including acute phase response markers, inflammation markers, growth factors, and adipokines. Specifically, MBDA includes the measurement of CRP, IL-6, serum amyloid A (SAA), TNF receptor 1 (TNFR1), epidermal growth factor (EGF), vascular endothelial growth factor A (VEGF-A), vascular cell adhesion molecule 1 (VCAM-1), matrix metallopeptidase 1 and 3 (MMP-1, MMP-3), chitinase-3-like-1 (CHI3L1; known also as YKL-40), resistin, and leptin levels. An elevated MBDA score divulges subclinical inflammation, regardless of clinical symptoms, and is correlated with increased recurrence risk [12]. Co-evaluation with positive APCA increases MBDA’s prognostic value, as it predicts more than 80% of relapses. A low MBDA score with a negative APCA is associated with less than a 13% chance for recurrence, allowing rheumatologists to taper or even stop b-DMARDs therapy [12]. Although the implementation of proteomics as a response index is helpful, particularly in relation to TNF blockers (etanercept and infliximab), a reliable prognostic model is yet to be established.

Epigenomics refers to heritable modifications in gene expression that play a crucial role in RA development, such as DNA methylation, histone modifications, and miRNA expression. Methylome analysis in whole blood samples revealed differences in the mononuclear cells of RA patients, which were hypomethylated compared to healthy controls [9]. These results were linked to DAS28 score, implying their significance in the early diagnosis of RA. Distinct methylation signatures were found in peripheral blood B-cells and T-cells supporting their potential role in classification, prognosis, and response to b-DMARDS [2,9,10,13]. Although DNA methylation is a more stable biomarker than gene expression and protein levels, its usefulness is limited by age, diet, smoke, alcohol consumption, and environmental factors. Nonetheless, the methylation status of synovial tissue precisely reflects the cellular diversity and enables molecular classification according to the distinct phenotypes of RA. During inflammation, T-cell function is differentiated among different methylation profiles, while a strong association between T-cell regulation and methylome analysis is revealed [14]. The synovium presents a wide and unique range of cell subpopulations that interfere with RA pathogenesis, indicating that its methylation levels could contribute to the establishment of innovative cell therapies [3,9,15,16]. The co-assessment of DNA methylation levels in synovium and multiplex proteomic analysis in serum samples might increase the prognostic value but entails invasive procedures and decreases the adherence rate. Hence, we pose the need to generate a blood multi-marker that combines proteomic, genomic, and methylome analysis and serves as a diagnostic, prognostic, and surveillance tool simultaneously.

Emerging methodologies in the flourishing field of molecular medicine appear to be the “passkey” for unlocking the pathobiology of RA, stratifying and monitoring patients, while permitting the parallel de-escalation of therapy when remission is achieved, or the revision of therapeutic strategy when multidrug resistance occurs. The incorporation of multiple genes, miRNAs, proteins, DNA methylation, and inflammation biomarkers in a multiplex panel raises evidence-based expectations for the “all in one” management of RA. Further investigation is necessary to define the biomarkers that should be included in order to diligently explore the heterogeneous nature of RA, introducing patients to the benefits of precision rheumatology. In this vein, *IJMS* reasonably devotes a significant part of its content to articles with a strong emphasis on evolving and challenging issues in the burgeoning field of personalized molecular medicine.
